# News media representations of women who kill their newly born children

**DOI:** 10.1007/s00737-021-01148-x

**Published:** 2021-06-13

**Authors:** B. Eisenwort, P. Fernandez Arias, C. M. Klier, B. Till

**Affiliations:** 1 Department of Pediatrics and Adolescent Medicine, Medical University of Vienna, Vienna, Austria; 2 Comprehensive Center Pediatrics, Medical University of Vienna, Vienna, Austria; 3 Monash Deakin Filicide Research Hub, Department of Social Work, Monash University, Melbourne, Australia; 4 Unit Suicide Research and Mental Health Promotion, Department of Social and Preventive Medicine Center for Public Health, Medical University of Vienna, Vienna, Austria

**Keywords:** Neonaticide, Media, Language, Linguistic Inquiry and Word Count (LIWCS), Verdict, News

## Abstract

This paper presents a first quantitative analysis of language in media reports of neonaticide and a comparative examination of language use within the reports. One thousand twenty-seven Austrian print media reports from 2004 to 2014 were retrieved; after exclusion, 331 were analysed using the Linguistic Inquiry and Word Count (LIWC) software. After a preliminary analysis, a comparative analysis was carried out between reports on the Graz case and all other cases. The preliminary analysis revealed that a majority of media reports were related to one repeat neonaticide case (Graz) despite not being clinically different from other cases identified for the same period. The comparative linguistic analysis shows some statistically significant differences relating to the domains of emotional words (less words of anxiety, sadness) and family and in the category of insight and certainty (more words). The unexpected media attention on the Graz case and the ensuing verdict, which was in contradiction with the Austrian infanticide act, might have been influenced by the way language was used by journalists and the media. The authors suggest guidelines on sensitive media reporting are required.

## Introduction

### Women who kill: neonaticide

For many, perhaps the single most notorious crime a woman can commit is killing her own children. Figures such as Medea continue to be part of the cultural inheritance that is passed on in Western cultures and, when these rare events take place, images of mothers who killed their children plague the media (Barnett 2006). However, the difference between what we imagine happening in these circumstances and the reality of the events is a considerable one. While women who kill do so in a variety of contexts and for different reasons, this paper focuses on women who kill their children and more specifically their newborns within the first 24 h of life. This form of child homicide is referred to as neonaticide and has been researched mainly from a sociolegal perspective.

Early research into neonaticide suggested the women were young, uneducated, single and poor (Resnick 1970). Recent scholarship presents a more complex and nuanced picture. As improvements in reproductive rights, contraception and sex education become more accessible, the risk factors and behavioural patterns associated to neonaticide no longer apply. Amon et al. (2012) identified that high fertility rates, high rate of negation of pregnancy, early childhood trauma and social unawareness of the pregnancy were the most important risk factors for neonaticidal women in their study. These risk factors were present even when the women were in stable relationships and lived with their partner or other children (Amon et al. 2012). In some 20% cases, severe mental illness is involved which may lead to a psychotic denial of pregnancy (Resnik 1970). Further analysis of the data comparing single and repeat neonaticide offenders, conducted by Klier et al. (2019), noted that repeat offenders were older and had a higher number of biological children who were living with them over their lifespan. However, for both single and repeat offenders, social unawareness of pregnancy remained high and was identified as a risk factor.

Despite the persistent popularity of crime stories in the media, stories about infanticide or neonaticide present challenges in terms of the complexity that needs to be conveyed to readers. Historically, what journalists have done to make these stories digestible for readers is use prevailing cultural myths to provide easy to understand explanations for the events (Barnett 2007). Stories of maternal filicide or infanticide are reported in sensational terms using lay accounts to provide context and explanations further entrenching common misconceptions (Barnett 2006). Reviews of media representations of women who kill have shown that women are systematically categorized into mad or bad and that this categorization shapes the legal responses of the justice system (Cavaglion 2008; Easteal et al. 2015).

In the Austrian media, for example, these women have often been labelled as “child murderers” (German: *Kindsmörderin*) and “coldblooded” (German: *kaltblütig*), and infanticide has often been described as “horrible act” (German: *schauderhafte Tat*) and “family drama” (German: *Familientragödie*). Very often, the newspaper article includes many details on how the baby was killed (e.g., “smashed skull”; German: *zertrümmerter Schädel*) or how the body was hidden (e.g., “embedded in concrete”; German: *einbetoniert*). For further details on the original study and the cases, outcomes and sentences, refer to Amon et al. (2020).

Media reporting can reflect and form cultural scripts and therefore they can also shape legal responses. Criminal proceedings themselves can be understood as a social rite that reinforces social cohesion and sets boundaries for its members in an authoritative style with a high degree of certainty concerning decisions and sentences (Tallgren 2013). But what are the linguistic features used in media reporting, which are responsible for shaping the social script of neonaticide and consequently shaping legal responses?

This paper presents an analysis of one noteworthy example—because of its extensive, negative and humiliating reporting—to shed light on the role of the media in reflecting and forming the cultural script of neonaticide in society. Cultural scripts consist of attitudes, evaluations and assumptions that are widely known and shared among people within a given speech community (Goddard 2006). Media reports are considered both to reflect and form cultural scripts (Ochs 1993; Eisenwort et al. 2014). Accordingly, journalist’s word-use is linked to social identities being communicated on one side and forming these identities on the other.

## Studying language

Language is the most common and reliable way for people to translate their internal thoughts and emotions into a format that others can understand. By the same token, use of a natural language provides important cues as to how people process this information. Certain cognitive mechanisms are indicative of more complex language alongside other word categories like prepositions, causal words, or words with more than six letters (Tausczik and Pennebaker 2010).

Corley and Wedeking (2014) show that as the level of certainty—one subcategory of cognitive mechanisms represented by words such as always, never, or exactly—in the Supreme Court’s opinion increases, the lower courts are more likely to positively treat the Supreme Court’s decision. Another subcategory of cognitive mechanisms is insight words such as to think, to know, acceptance, to answer and to mean. Li et al. (2019) studying the influence of reviews on consumer purchase behaviour found that one of the linguistic features characterizing the writing style of top reviewers, compared with ordinary reviewers, is an analytical writing style. Their reviews are evaluated as more helpful because they involve more analytical thinking patterns alongside other features.

In Ritter et al. (2013) study exploring text messages on Twitter from Christians and atheists, the latter showed an analytic writing style characterized by more insight words which predicted decreased happiness. They found more positive and fewer negative emotions in text messages from Christians. This relation was partially mediated by linguistic markers of social connection and thinking style. Christians more often mentioned social processes that suggest stronger relationships and support networks. Pressman and Cohen (2007) consider social words as an indirect measurement of social relationship, too.

Tentative wording, which includes words like “maybe” or “perhaps”, is another subcategory of cognitive mechanisms. It characterizes the cautious writing style of an author uncertain about the circumstances. The language that people use to discuss an event can reveal something about the extent to which a story may have been established or is still being performed. When people are uncertain about their topic, they use tentative language (Tausczik and Pennebaker 2010). Eisenwort et al. (2014) showed that in media reporting on female suicide, more tentative wording is used compared with reports on male suicide. Female suicidal behaviour is constructed as more extraordinary than male suicidal behaviour, and this is consistent with the finding that males are overrepresented in fatal suicidal behaviour.

Black et al. (2016) studied the role of emotion in language in the US Supreme court. In the USA, the legal brief is a primary vehicle by which lawyers seek to persuade appellate judges. Their hypothesis was that a judge would be less likely to vote for a party whose brief employs more emotional language. To test this, they analysed 1766 briefs to the Supreme Court justice with LIWC. They found that parties who use less emotional language are likely to win a justice’s vote. This means that writers using objective language can enhance their credibility. In summary, a writing style characterized by more certainty and insight words but fewer emotion words characterize an analytic writing style which makes messages more credible and credibility is a key component in persuasion (Black et al. 2016).

## The Graz case

In 2005, a neonaticide case was detected by a neighbour and when investigated by the police, a total of four dead newborns were found. This case stood out because of the extensive media coverage and the negative and humiliating reporting present. In addition to the court procedures, the misogynistic style was echoed by the experts (forensic and obstetric), the jury, the judge and federal prosecutor. From a clinical and forensic viewpoint, this case resembled all other neonaticide cases in the same jurisdiction in a 10-year period (Klier et al. 2019).

The mother was accused of having killed the newborns purposefully to take revenge on her partner as he had told her he did not want any more children (he had 3 children from his marriage with another women). The forensic expert argued that she could not have denied pregnancy more than once, that she must have learned from the first pregnancy and therefore this was not a neonaticide case. Moreover, he saw her age at the time of the deaths and that she had several pregnancies as a reason for exclusion for having committed a neonaticide and she was charged with murder and given a lifelong sentence despite a neonaticide law in Austria limiting prison terms from 1 to 5 years in such cases.

Her partner was charged for not having identified her pregnancies as it was deemed impossible that he did not know anything about her pregnancies and got a sentence of 15 years. He was the only male partner in 20 years and 55 cases who was charged in a neonaticide case which is, by definition, a female crime (Amon 2020). Experts were puzzled by the media attention this one specific case received and they refer to this case even to this day as the “Graz case”, after the city were the neonaticides and the court proceeding took place. The question arises then, is it possible that the language in the media reporting may have been different in this case compared to other neonaticide cases.

This paper presents an analysis of one noteworthy example—because of its extensive, negative and humiliating reporting—to shed light on the role of the media in reflecting and forming the cultural script of neonaticide in society. Cultural scripts consist of attitudes, evaluations and assumptions that are widely known and shared among people within a given speech community (Goddard 2006). Media reports are considered both to reflect and form cultural scripts (Ochs 1993; Eisenwort et al. 2014). Accordingly, journalist’s word-use is linked to social identities being communicated on one side and forming these identities on the other.

## Method

### Hypotheses

What features characterize the writing style in the media coverage of the Graz case?

Based on the results of Corley and Wedeking (2014) who argue that certainty or “authoritativeness” is a basic but unrecognized tool to policy makers, we assumed that media reporting in the Graz case is more authoritative than in other neonaticide cases.

- H1: The wording of the Graz case contains more certainty words than wording of other neonaticides.

Based on Li et al. (2019), we assume that an analytic writing style has more power to influence the reader. Consequently, we think that media coverage of the Graz case—which is characterized by more insight words—inspires the reader to process information in a negative and humiliating way compared with media coverage of other neonaticide cases.

- H2: The wording of the Graz case contains more insight words than wording of other neonaticides.

Regarding the influence of emotional language, Black et al. (2016) could show that judges are more likely to vote for parties whose briefs are written in an objective language with few emotion words. Briefs using emotional language are less likely to persuade the Supreme Court. We conclude that emotion words can decrease credibility in a context where official language is needed.

- H3: The wording of the Graz case contains fewer emotion words referring to sadness and anxiety than wording of other neonaticides.

According to many authors like Pressman and Cohen (2007), social words are an indirect measurement of social relationships, and in Ritter’s study, Christians mention social processes more often suggesting stronger relationships and support networks and more positive and fewer negative emotions (Ritter et al. 2014). Because of the extensive media coverage in a negative and humiliating way, we assume that the perpetrator is not described within her social network but is described as isolated and in a state of emergency.

- H4: The wording of the Graz case contains fewer words referring to family than wording of other neonaticides.

### Included media reports

We analysed Austrian print media reports published between December 16, 2004, and December 16, 2014 from the Austrian Press Agency that included the word “Babymord” (killing of a baby), “Kindesmord” (neonaticide, child murder), or “Kindestötung” (infanticide). The majority of the newspapers were daily newspapers. All national newspapers (e.g., *Kronen Zeitung*, *Kurier*) as well as those regional newspapers with the highest circulation in Austria (e.g., *Kleine Zeitung*, *Tiroler Tageszeitung*) were included in the analysis. The target audience of these newspapers was highly diverse in terms of socio-demographics and political orientation. Our search yielded 1027 articles. We excluded 696 articles, because the focus was on another topic: 228 focused on fictional stories, 11 articles described war victims and their children, one article mentioned child murder as opposed to murder of an adult, 85 articles were duplicates, 81 articles were letters from readers, 75 articles were texts from Austrian press agency (not newspaper articles), nine articles focused on murdering of pets, and 206 articles focused on physical abuse of children. In total, 331 articles fulfilled the inclusion criteria and were included in the final data analysis (see Table 1).

### Data analysis

The Linguistic Inquiry and Word Count (LIWC) software (Pennebaker et al. 2001) was used to analyse the wording of the texts. LIWC is a word count-based text analysis program, which uses distinct word categories with more than 2300 words or word stems. LIWC operates by comparing all words in a given text to a dictionary and by counting the number of words in this text that fall into specific word categories relative to the total number of words (text length), i.e., the percentage of all of the words in that text (Pennebaker et al. 2001). We used the German translation of LIWC by Wolf et al. (2008). The German LIWC categories have been shown to have high equivalence to their English counterparts based on two studies using 122 bilingual text units and 104 emails.

The goal of LIWC is to use objective linguistic data to gain information about an individual’s cognitive processing, including attentional focus, emotionality and thinking styles. Exploring media reports with LIWC means shedding light on cultural scripts of a society. In our study, our basic research question is whether there are differences between the language of the Graz case and other cases of neonaticides in Austrian newspapers. Regarding the articles, there are two different categories of text differing in writing style: articles on the case itself on one hand and articles about the trial/prosecution of the perpetrators on the other hand. Therefor the scores for all LIWC categories were subjected to a case (case in Graz vs. other cases) × coverage (case coverage vs. trial coverage) analysis using generalized linear models. Because our residuals were not normally distributed, we conducted generalized linear models with gamma distributions (Venables and Ripley 2002).

## Results

Of the 331 newspaper articles included in the statistical analyses, 226 articles (68.3%) reported on the infamous case in Graz, whereas the remaining 105 articles (31.7%) were on other neonaticides. Furthermore, 147 articles (44.4%) covered the respective case (case in Graz: *n* = 59; other cases: *n* = 88), whereas 184 articles (55.6%) were classified as coverage of the trial (case in Graz: *n* = 167; other cases: *n* = 17).

The statistical analysis results are presented in Table 1 (see below). Wording indicating certainty and insight were more common in newspaper articles on the case in Graz than in newspaper articles covering other neonaticides. There were also more words related to friends in the articles on the case in Graz, but the significant interaction effect with coverage indicates that this effect may be based on the high proportion of trial coverage among the articles on the case in Graz. In contrast, wording related to sadness, anxiety and family were less common in the articles on the case in Graz than in those of other cases.

**Table 1:** Descriptive statistics stratified by case and type of news coverage and findings from generalized linear models for use of words in newspaper articles on neonaticide (*n* = 331)^a^

Variable	Case in Graz (*n* = 226)	Other cases (*n* = 105)	Case coverage (*n* = 147)	Trial coverage (*n* = 184)	Case (Graz vs. others)	Coverage (case vs. trial)	Case × coverage
	*M* (95% CI)	*M* (95% CI)	*M* (95% CI)	*M* (95% CI)	χ^2^	χ^2^	χ^2^
Emotion							
Anger/aggression	0.24 (0.19–0.29)	0.36 (0.25–0.48)	0.32 (0.23–0.41)	0.24 (0.18–0.30)	1.46	1.38	2.18
Sadness	0.20 (0.15–0.24)	0.38 (0.27–0.49)	0.33 (0.25–0.41)	0.20 (0.14–0.26)	8.78^**^	0.56	0.11
Anxiety	0.16 (0.12–0.20)	0.31 (0.19–0.43)	0.25 (0.17–0.33)	0.17 (0.12–0.23)	10.41^**^	0.18	1.24
Heterogeneity							
Insight	2.21 (2.04–2.39)	1.78 (1.58–2.00)	1.90 (1.70–2.09)	2.22 (2.03–2.41)	4.26^*^	0.18	0.43
Tentative	0.49 (0.41–0.57)	0.59 (0.46–0.71)	0.57 (0.45–0.69)	0.48 (0.40–0.56)	1.31	0.02	1.23
Certain	1.01 (0.90–1.13)	0.71 (0.56–0.86)	0.77 (0.64–0.90)	1.04 (0.90–1.17)	5.02^*^	0.42	1.43
Social processes							
Social	11.35 (10.88–11.82)	11.00 (10.32–11.68)	10.49 (9.94–11.05)	11.83 (11.31–12.35)	0.65	7.09^**^	1.12
Family	1.74 (1.55–1.94)	2.57 (2.29–2.86)	2.33 (2.08–2.57)	1.75 (1.54–1.96)	9.17^**^	1.02	0.00
Friends	0.50 (0.42–0.59)	0.27 (0.17–0.36)	0.31 (0.23–0.39)	0.53 (0.43–0.63)	9.75^***^	0.17	6.96^**^

^a^Values are means (*M*) with 95% confidence intervals (95% CI) given in parentheses as well as χ^2^ values from generalized linear models with gamma distributions (^*^ *p* < 0.05; ^**^ *p* < 0.01; ^***^ *p* < 0.001; two-tailed). Each word of a linguistic subcategory was counted in each article and arithmetic means of percentages of these counts per total word count were separately calculated and compared with regard to case (case in Graz vs. other cases) and type of news coverage (case vs. trial coverage)

There were no differences regarding the LIWC categories *Anger/Aggression*, *Tentative* and *Social* between the two groups. Table 1 displays the means and corresponding 95% confidence intervals for all outcome variables for all types of newspaper articles and gives an overview of the results of the generalized linear models. Based on these results, all four hypotheses were supported. Of note, newspaper articles covering the trial of a neonaticide contained more “social words” than articles reporting on the case per se. This was independent of whether the articles were on the case in Graz or other neonaticide cases.

## Discussion

This study is the first to present quantitative data analysis on neonaticides portrayed in Austrian news media. The results show that not only is media interest high when a case of neonaticide occurs, but also that linguistic analysis can shed light on the discourse of the media. The number of media reports concerning the Graz case (68.3% of the total of reports in 10 years) suggests that this intensity may have influenced the outcome unfavourably. When the number of reports on the trial was counted, more than 90% were from the Graz case (167 vs. 17 cases). The question of why this case was referred to as exceptional and other repeat cases were not at all mediatized is difficult to answer. Not every crime gets the attention of the media when other important events happen at the same time or local journalists do not get the information from police or court.

The writing style of the media regarding the Graz case is characterized by the use of more certainty and insight words compared with reporting on other neonaticide cases. More certainty words characterize an authoritative writing style (Corley and Wedeking 2014). More insight words characterize an analytic writing style. This means that, to our point of view, it may be that authoritativeness together with an analytic writing style creates the public perception of the case as extraordinary, despite the fact that from a clinical and forensic viewpoint, it resembles all other neonaticides in a 10-year period (Klier et al. 2019). Wording related to sadness and anxiety was less common in the articles on the case in Graz than in those of other cases. Based on Black et al. (2016), objective language with fewer emotion words can enhance credibility. Finally, less wording on family shows a perpetrator in isolation.

Similarly, the Graz style of reporting, more certainty and insight words but fewer emotion words and words related to family, echoes the language used in criminal trials, which sets boundaries for its members in an authoritative style with a high degree of certainty concerning decisions and sentences (see Black et al. 2016). Gurevich (2008) states that “maternal trials of crime against children could be perceived as expressions of societal needs for cohesion and boundary making” (p. 516). This would explain why the writing style of the Graz case had a strong persuasive potential. Further, it would also reflect and influence the public perception of this case as special and would provide a context for the unusually harsh sentence. Reporting of filicide trials, especially neonaticide, often lack sensitivity and scientific understanding of the phenomenon and can form a cultural script that promotes prejudice (Tallgren 2013). While this paper shows that there may be differences in the reporting between neonaticides, a clear outcome is that guidance on sensitive media reporting is warranted.

Further, given the involvement of medical professionals as expert witnesses within the criminal justice systems of most countries, it is important that these medical professionals have a better understanding of the interrelated nature of the work they may be performing. This paper also hopes to challenge the unconscious biases that heteronormative social structures have created. The belief that women ought to know when and if they are pregnant, that they will take to mothering in natural and unproblematic ways and that they would never harm their children continues to negatively impact women (Fernandez Arias et al. 2019). Moreover, bias has been known to have a direct impact on maternal health care (Khera et al. 2014; Omeish and Kiernan 2020; Saluja and Bryant 2020).

The present study had several limitations. First, it remains unclear if all news articles on neonaticides in Austria between 2004 and 2014 were retrieved. Relevant articles may not have been identified by our approach. Second, while duplicates were excluded from analysis, some articles were very similar to each other. This was particularly the case, when articles were used for different issues of the same newspaper or when they were based on the same press release. Lastly, although the method we used can be found in many studies, most relate to another topic and therefore cannot be used for explaining our results.

## Conclusion

There remains a long way in achieving responsible reporting of women who kill their children. Thus far, women are systematically categorized into mad or bad and this categorization shapes the legal responses of the justice system. This paper is a first linguistic study using a quantitative approach. The results suggest that media portrayals may influence criminal proceedings and verdicts. Recommendations for sensitive media reporting must be developed.

## Data Availability

Data on request.

## References

[CR1] Amon S, Putkonen H, Weizmann-Henelius G, Almiron MP, Formann AK, Voracek M, Eronen M, Yourstone J, Friedrich M, Klier CM (2012). Potential predictors in neonaticide: the impact of the circumstances of pregnancy. Archives of women's mental health.

[CR2] Amon S, Klier CM, Putkonen H, Weizmann-Henelius G, Fernandez Arias P (2020). Neonaticide in the courtroom – room for improvement?. Conclusions Drawn from Austria and Finland's Register Review Child Abuse Review.

[CR3] Black RC, Owens RJ, Wedeking J, Wohlfarth PC (2016). US Supreme Court opinions and their audiences.

[CR4] Barnett B (2006). Medea in the media: narrative and myth in newspaper coverage of women who kill their children. Journalism.

[CR5] Barnett B (2007). The wounded community: mother-blaming in journalistic accounts of maternal infanticide Media report to women.

[CR6] Cavaglion G (2008). Bad, mad or sad?. Mothers who kill and press coverage in Israel Crime, Media, Culture: An International Journal.

[CR7] Corley PC, Wedeking J (2014) The (dis)advantage of certainty: the importance of certainty in language law & society review 48:35-62 doi:10.1111/lasr.12058

[CR8] Easteal P, Bartels L, Nelson N, Holland K (2015). How are women who kill portrayed in newspaper media?. Connections with social values and the legal system Women's Studies International Forum.

[CR9] Eisenwort B, Till B, Hinterbuchinger B, Niederkrotenthaler T (2014) Sociable, mentally disturbed women and angry, rejected men: cultural scripts for the suicidal behavior of women and men in the Austrian print media sex roles 71:246-260 doi:10.1007/s11199-014-0395-3

[CR10] Fernandez Arias P, Yoshida K, Brockington IF, Kernreiter J, Klier CM (2019). Foetal abuse Arch Womens Ment Health.

[CR11] Goddard C (2006). Ethnopragmatics : understanding discourse in cultural context.

[CR12] Gurevich L (2008). Patriarchy?Paternalism? Motherhood discourses in trials of crimes against children. Sociol Perspect.

[CR13] Khera R, Jain S, Lodha R, Ramakrishnan S (2014). Gender bias in child care and child health: global patterns. Archives of disease in childhood.

[CR14] Klier CM, Amon S, Putkonen H, Fernandez Arias P, Weizmann-Henelius G (2019). Repeated neonaticide: differences and similarities to single neonaticide events. Archives of Women's Mental Health.

[CR15] Li S, Modi P, Wu M-S, Chen C-H, Nguyen B (2019) Conceptualising and validating the social capital construct in consumer-initiated online brand communities (COBCs) Technological forecasting & social change 139:303-310 doi:10.1016/j.techfore.2018.11.018

[CR16] Ochs E (1993). Constructing Social Identity: A Language Socialization Perspective Research on language and social interaction.

[CR17] Omeish Y, Kiernan S (2020) Targeting bias to improve maternal care and outcomes for Black women in the USA EClinicalMedicine 27 10.1016/j.eclinm.2020.100568 PMC7599311 33150330

[CR18] Pennebaker JW, Francis ME, Booth RJ (2001). Linguistic inquiry and wordcount -LIWC 2001.

[CR19] Pressman S, Cohen S (2007). Use of social words in autobiographies and longevity. Psychosomatic Medicine.

[CR20] Resnick PJ (1970). Murder of the newborn: a psychiatric review of neonaticide. The American journal of psychiatry.

[CR21] Ritter RS, Preston JL, Hernandez I (2013). Happy tweets: christians are happier, more socially connected and less analytical than atheists on twitter. Social Psychological and Personality Science.

[CR22] Saluja B, Bryant Z (2020) How implicit bias contributes to racial disparities in maternal morbidity and mortality in the United States Journal of Women's Health 10.1089/jwh.2020.8874 33237843

[CR23] Tallgren I (2013). The Durkheimian Spell of International Criminal Law?. Revue interdisciplinaire d'études juridiques.

[CR24] Tausczik YR, Pennebaker JW (2010). The psychological meaning ofwords: LIWC and computerized text analysis methods. J Lang Soc Psychol.

[CR25] Venables WN, Ripley BD (2002). Modern applied statistics with S.

[CR26] Wolf M, Horn AB, Mehl MR, Haug S, Pennebaker JW, Kordy H (2008). Computergestützte quantitative Textanalyse: Äquivalenz und Robustheit der deutschen Version des Linguistic Inquiry and Word Count. Diagnostica.

